# Increased Fat-Free Body Mass and No Adverse Effects on Blood Lipid Concentrations 4 Weeks after Additional Meat Consumption in Comparison with an Exclusion of Meat in the Diet of Young Healthy Women

**DOI:** 10.1155/2011/210930

**Published:** 2011-06-14

**Authors:** Klaus J. Petzke, Susen Lemke, Susanne Klaus

**Affiliations:** Group of Stable Isotopes and of Energy Metabolism, German Institute of Human Nutrition in Potsdam-Rehbruecke (DIfE), Arthur-Scheunert-Allee 114-116, 14558 Nuthetal, Germany

## Abstract

*Aims*. To investigate whether changes of meat consumption can affect body composition and laboratory parameters in healthy, normal weight, young women without the aim to reduce body weight. *Research Design and Methods*. Women volunteered to eat low-fat meat in addition to their habitual diet (M) or to exclude meat products from their diet (NOM). After 4 weeks M and NOM were crossed over between subjects. Changes in nutrient intake, morphometrics and plasma parameters were compared during M and NOM. *Results*. Daily protein intake (means ± SD) was 2.25 ± 0.35 (25.2% of energy) and 1.15 ± 0.26 g/kg (14.0% of energy) during M and NOM, respectively. Fat-free body mass (FFM) increased during M (0.7 ± 1.0 kg, *P* = .02) and decreased during NOM (−0.8 ± 0.8 kg, *P* = .003). Body fat mass was unchanged. Concentrations of total cholesterol (−7%), LDL-cholesterol (−8%), and glucose (−4%) deceased significantly after M. Fasting glutamine concentrations were decreased by M and increased by NOM. *Conclusions*. Additional meat intake can increase FFM without adverse effects on blood lipid concentrations. Long-term studies are required. Urinary excretion of 3-methylhistidine could represent a biomarker for meat protein consumption.

## 1. Introduction

High-protein diets are suggested to increase satiety, to benefit fat oxidation, to reduce energy efficiency during overfeeding, and to contribute to a better conservation of fat-free body mass (FFM) which can support diet therapies of biomedical problems such as obesity or loss of FFM [1–4]. For the majority of the population, a practical means of increasing the intake of protein of high biological value is the introduction of lean meat into each meal [5]. Moreover, meat is one of the main animal protein sources of habitual diets in Germany (~25% of total protein intake; median meat intake of 65 g/d for 25–34 y old women) [6]. However, benefits and risks of high-protein and (or) high-red-meat-containing diets are controversially discussed and there is a lack of information about long-term effects [7–9]. Recent epidemiological studies suggest that a high consumption of red meat may be linked with diabetes and some types of cancer [10–12]. In addition, Micha et al. [13] concluded in a systematic review and meta-analysis that processed meat, but not red meat, is associated with a higher incidence of cardiovascular diseases and diabetes mellitus. Nonetheless, the results of a recent large follow-up study indicate that meat consumption is positively related to weight gain in men and women and in normal-weight and in overweight subjects suggesting that after all a decrease in meat consumption may improve weight management [14]. In this sense, it is important to investigate the effects of changes in the consumption of meat in the etiology of different pathologies. In our study we aimed to compare the outcomes of an adaptation to an additional intake of meat with those of an omission of all meat products on selected anthropometric and laboratory parameters. Young, healthy, normal weight women who took part in this crossover study volunteered to eat low-fat meat (200 g lean pork fillet corresponding to ~42 g protein) in addition to their habitual diet or to exclude all meat products from their diet for 4 weeks, respectively. Subjects were supposed not to reduce body weight for a better discrimination of possible effects of high-protein (-meat) intake from other metabolic effects due to weight reduction.

## 2. Subjects and Methods

### 2.1. Subjects and Study Design

Fourteen female subjects were recruited among students of the Institute of Nutritional Sciences at the University of Potsdam. Inclusion criteria were good health, age between 20 and 30 years old, a habitual protein intake of about 1 g/kg/d, and no excessive intake of semiluxury food. Exclusion criteria were infectious, metabolic, and gastrointestinal diseases, food intolerances, eating disorders, pregnancy and lactation, extreme forms of dieting, and regular usage of drugs. After having received verbal and written information about the study, all subjects gave written consent. The study protocol was approved by the Ethics Committee of the University of Potsdam in accordance with the Helsinki declaration (decision 8/19 of November 18th, 2004).

The study had a randomized, crossover design, where 4 weeks of additional intake of exclusively low-fat pork fillet (M) and an exclusion of meat and meat products (NOM) was examined, respectively. Baseline measurements were executed. After this seven of the subjects started the first dietary intervention period M (group A). Seven other subjects started with NOM (group B). After four weeks the interventions M and NOM were crossed over between subjects. During period M, participants were asked to eat the 200 g of lean pork fillet per day in addition to their usual diet. Pork fillet was bought by participants from local supermarkets and butcher's shops and costs were compensated. The origin of pork fillet was recorded. Participants were allowed to distribute the additional meat intake during M over the whole day. However, it was recommended not to be eaten together with the usual meals. This was done to ensure that the meat was eaten additionally to the habitual subjects' diet and that it was not used only for a replacement of their usual portions. Individual portions were reported in a protocol. During NOM participants were asked to abstain from consumption of all meat and meat products but were allowed to eat eggs and dairy products.

### 2.2. Data and Sample Collection

Dietary intake information was assessed by a semiquantitative and self-administered 4-d food record from Sunday to Wednesday prior to the study and during the fourth week of each intervention period [15]. The record was assigned by staff and subjects were instructed to record their entire food intake at the time of consumption. The food record as well as an accompanying data entry and nutrient calculation software program are available for download including a complete documentation of food coding (http://www.dife.de/). The coding of nutrient intake (including individual amino acids) was carried out on the basis of the German Food Code and Nutrient Data Base BLS II.3 (Federal Institute for Health and Protection of Consumers and Veterinary Medicine, 1999). 

Subjects visited the institute between 0800 and 1000 h after an overnight fast at baseline and after 4 wks of each intervention period for anthropometric measurements and for sample collection. Blood samples were drawn into Li-heparinized tubes (Monovette, Sarstedt AG and Co., Nürnbrecht, Germany) which were placed on ice within 10 min. Plasma was separated by centrifugation (10 min, 4°C, 2,000 × g), frozen in liquid N_2_ within 30 min of blood collection, and stored at −80°C before analysis. 24-hour collections of urine were made at baseline and within week 3 of each intervention period. Nitrogen excretion was measured in these samples.

### 2.3. Anthropometrics, Body Composition, and Laboratory Procedures

Anthropometric measures were obtained by trained personnel while subjects wore no shoes and only light underwear. Body weight was assessed on an empty bladder using a digital scale to the nearest 100 g (Soehnle, Murrhardt, Germany). Body height was measured with a flexible anthropometer to the nearest 0.1 cm (Silber & Hegner, Zürich, Switzerland). Body mass index (BMI) [weight (kg)/height^2^ (m^2^)] was calculated. Body fat mass and fat-free body mass (FFM) were measured by air displacement plethysmography (BodPod, Life Measurement, Inc., Concord, CA) according to the manufacturer's recommendations. 

Concentrations of cholesterol, triacylglycerol, glucose, and insulin were measured using colorimetric or enzymatic standard methods. Nitrogen content of urine was determined using an elemental analyzer (intra-assay CV < 3%) (EA 1108, Fisons Instruments, Rodano, Italy). Amino acid and urea concentrations in plasma and urine were analyzed by ion-exchange chromatography as reported previously (intra-assay CV < 5%) [16]. The determination of total homocysteine (THC) was performed by GC-MS (intra-assay CV < 5.5%) (Varian Chromatography systems Walnut Creek, CA, coupled with SSQ 710, Thermo Finnigan, Bremen, Germany) on a DB-5MS column (30 m × 0.25 mm ID, 0.25 *μ*m film thickness; J&W Scientific, Folsum CA) using DL-[3,3,3′,3′,4,4,4′,4′-D_8_]homocystine (98%) (CIL, Andover, MA) as an internal standard [17].

### 2.4. Statistical Analyses

All data were tested for normal distribution with the Shapiro-Wilk test for normality. One value of FFM which was obviously an outlier possibly due to false data input was excluded from calculations. Descriptive analysis includes means ± SD. Mean values are presented for baseline, M, and NOM (*n* = 14). The means after M and after NOM were tested for significant differences by dependent (paired samples) *t*-test. In addition, changes were calculated between subject-specific values after either M or NOM in relation to values before M or NOM, namely, baseline or end of first intervention period. The significance of the resulting effect of M or NOM calculated as change of individual values was computed using the 2-tailed one sample *t*-test with zero as hypothetical mean value. The relationships between variables were estimated by Pearson correlations (anthropometric and biochemical data against dietary data). Regression analysis was used to evaluate the relationship between dietary data and measured data. Standardized regression coefficients were tested by *t*-test for deviation from the null hypothesis. The significance threshold was set at *P* = .05. We used SPSS for Windows (version 14.0.1, SPSS GmbH Software, Munich, Germany) or WinSTAT (version 1999.2, R. Fitch software, Staufen, Germany) for statistical evaluations.

## 3. Results

### 3.1. Subjects

Baseline characteristics of the subjects are presented in Table 1. The dietary interventions were well tolerated by all subjects. Biochemical values at baseline and after intervention periods were found to be within normal ranges (Table 3). The primary outcome measurements were not significantly different at baseline between 7 subjects starting first intervention period with either M or NOM.

### 3.2. Dietary Intervention

Dietary macronutrient and energy intake were reported in Table 2. At baseline the subjects' diets provided energy in the form of 17% protein, 32% fat, and 51% carbohydrate. Furthermore, throughout period M, the daily protein consumption was higher by 0.85 and 1.1 g/kg as compared to baseline and NOM, respectively [18]. Throughout M and NOM subjects consumed 8% more and 3% less energy in the form of protein as compared to baseline, respectively. The changes in protein consumption were based on the modifications of meat protein intake. Consequently, the values of urinary urea and 3-methylhistidine (3MH) excretion were significantly higher during M than during NOM (Table 3). The protein consumption was positively correlated with urinary urea and with 3MH excretion rate (*r* = 0.733, *P* = 1.5 × 10^−8^ or *r* = 0.827, *P* = 7.2 × 10^−12^, resp.). Furthermore, an endogenous 3MH excretion of *∼*90 *μ*mol/d was computed relating the values of daily 3MH excretion of all participants against the animal protein intake at baseline, during M and during NOM based on 4-d food records (*y* = 0.0226*x* ^2^ + 0.962*x* + 91.1, *r* = 0.854). 

The total fat consumption was significantly higher during M as compared to NOM. However, in terms of energy percentage, fat contribution was not significantly different. The total carbohydrate consumption was not significantly different between M and NOM. However, carbohydrates provided about 10% less energy throughout M as compared to NOM. The mean total energy intake was significantly higher during M by about 290 kcal/d compared to NOM.

### 3.3. Body Composition and Laboratory Measurements

**Figure 1:**
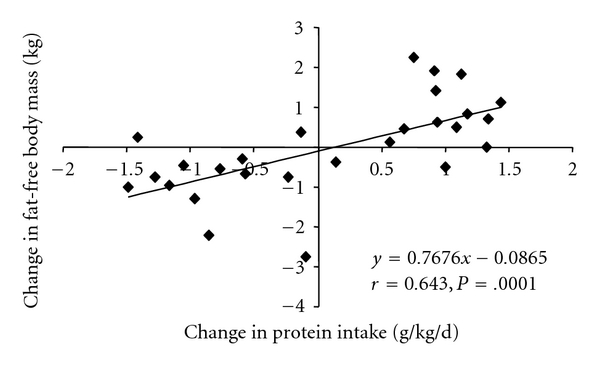
Combined plot showing the relationship between changes in protein intake and changes of fat-free body mass (FFM) of 14 young healthy women after consumption of diets for 4 wks containing either additional meat (M, 200 g pork fillet/d) or exclusion of all meat products (NOM). Individual differences have been computed for each of the 14 subjects after each 4 weeks period relative to previous period, namely, baseline or end of first intervention period. Calculations of protein intake were based on 4-d food records. One value of FFM which was obviously an outlier was excluded.

The BMI was significantly higher after M compared to NOM (Table 1). The BMI increased during M (+0.24 ± 0.30 kg/m^2^, *P* = .027) and decreased during NOM (−0.28 ± 0.23 kg/m^2^, *P* < .1 × 10^−4^). However, the significant increase in body weight by about 0.7 kg during M (*P* = .02) and the decrease in body weight by about 0.8 kg during NOM (*P* = .7 × 10^−4^) was based exclusively on changes of FFM. In addition, there was a positive correlation of changes in total protein intake with changes in BMI (*r* = 0.704, *P* = .1 × 10^−5^) and FFM (Figure 1).

Interestingly, fasting concentrations of total cholesterol, LDL cholesterol, and glucose were not significantly different between M and NOM (Table 3). However, the means of individual changes were significantly lower (*P* < .05) after M by 7%, 8%, and 4%, respectively (Table 4). Furthermore, the changes in total protein consumption during both M (increase) and NOM (decrease) were negatively correlated with individual changes in fasting concentrations of glucose (*r* = −0.356, *P* = .031) and total cholesterol (*r* = −0.336, *P* = .04) (data not shown). The fasting mean THC concentrations in plasma were also not significantly different between M and NOM and were ~10 *μ*mol/L (Table 3). As compared to baseline the mean THC concentrations were higher after both M and NOM by ~3 *μ*mol/L and the increases were significant (*P* < .001, Table 4). There was no significant correlation between changes in total protein intake and changes of fasting THC concentrations (*r* = 0.217, *P* = .08). However, there revealed a negative correlation between changes in total protein intake during both M and NOM and individual changes in plasma glutamine concentrations (*r* = −0.362, *P* < .03) or changes in glutamine to cystine ratios (*r* = −0.518, *P* < .003) (data not shown) which was suggested to be a marker of skeletal muscle catabolic state [19]. This correlation was based predominantly on significant negative (−41 ± 74 *μ*mol/L) and positive (+38 ± 60 *μ*mol/L) individual changes of fasting glutamine concentrations after M and NOM, respectively. Nevertheless, the mean fasting concentrations of indispensable amino acids lysine and valine were significantly higher after M compared to NOM (Table 5). 

## 4. Discussion

Previous studies on the health effects of high-protein diets were mostly performed within the context of weight loss diets in which it may be difficult to discriminate between metabolic effects due to weight loss or due to protein consumption by itself. Here we present the results of a study in healthy, normal-weight women without the aim to reduce body weight. The primary finding was that adverse effects were not detected on body composition, concentrations of blood lipids, insulin, glucose, and THC measured after a 4 weeks dietary intervention with additional meat intake as compared to an exclusion of meat products which considerably increases or decreases the habitual daily animal protein consumption, respectively. The results have shown that after 4 weeks additional meat consumption FFM was higher in young normal-weight woman without changes in body fat content. The stability of body fat content during M is remarkable as the energy intake is higher compared to NOM. This result may be explained by higher energy expenditure in addition to increased fat oxidation following consumption of high-protein meals [20, 21].

Although, there are still no standard definitions for high-protein diets [8] we propose that the daily protein intake of about 1.4 g/kg body weight (17% of energy) at baseline in our healthy female subjects may be defined as relatively high compared with 0.8 g/kg as currently recommended in dietary allowances to exclude protein deficiency [18]. During M subjects consumed more than 2 g/kg/d of protein (25% of energy). During NOM the mean daily protein consumption declined to 1.15 g/kg (14% of energy), but it was still higher than 0.8 g/kg. The modifications of protein intake during M or NOM were clearly achieved by increases of meat consumption or the exclusion of all meat products, respectively (Table 2). This was reflected in blood levels and in excretion rates of urea which confirmed the good compliance of our study group. 

In addition, we could show that blood levels and excretion rates of 3MH may characterize the extent of meat consumption. 3MH is mainly a constituent of muscle and meat proteins. It is therefore possible to be used as a tool to measure muscle protein breakdown after consumption of meat-free diets because of its quantitative excretion in urine. The endogenous 3MH excretion rate of young woman was reported to be ~100 *μ*mol/d [22]. This corresponds with our results showing ~90 *μ*mol/d when the function between 3MH excretion and animal protein intake was extrapolated. An increase in meat intake was shown to raise the urinary 3MH excretion rate in a dose-dependent manner [23]. This is in line with our results showing raised 3MH excretion rates when the proportion of animal protein intake is higher which is based on changes in meat consumption. Thus, we assumed that the urinary 3MH excretion promises to be a reliable biomarker for meat intake provided that further validating studies are performed [24].

A better preservation of lean body mass during high-protein diets has been reported in studies aimed to reduce body weight. This result was primarily ascribed to higher rates of net protein synthesis, increased accretion of tissue protein, and restrained body protein breakdown [25–27]. Campbell et al. [28] reported greater gains in FFM and skeletal muscle mass in response to resistance training in older men consuming a meat-containing diet as compared with lacto-ovo vegetarian diet. In analogy, older women can better preserve their muscle mass introducing animal protein into their diet [29]. Tan et al. [30] have reported lower protein oxidation rates when meat-containing high-protein test meals were consumed compared with dairy or soy containing meals indicating protein sparing effects with meat. Finally, ingestion of lean beef servings were shown to increase all plasma concentrations of indispensable amino acids and the fractional skeletal muscle protein synthesis rates in healthy persons compared to premeal periods [5]. We did not measure parameters of protein turnover. However, based on the average increase of FFM of about 0.7 kg (~0.14 kg protein) during M it can be calculated that additionally ~4.5 g of protein were retained daily in our subjects. Therefore, we speculate that the additional consumption of lean meat as a source of high biological value protein (and other essential nutrients) can enhance FFM which may have consequences, for example, in alleviating biomedical problems of the metabolic syndrome.

There is an ongoing discussion about pathogenic roles of amino acids, for example, in the development of insulin resistance [7–9, 25]. We did not detect considerable differences in fasting plasma amino acid concentrations between M and NOM (Table 5) which indicates that amino acids even when high-protein diets such as M are consumed effectively can be disposed by postprandial catabolic processes. However, shortly after high-protein containing meals postprandial amino acid concentrations may rise substantially. This may induce an increase in protein balance and, therefore, can enhance FFM after long-term exposures [26, 31]. However, we find it noteworthy, that the fasting glutamine concentrations in plasma were lower with increasing protein intake which may have long-term consequences as this amino acid has important metabolic functions not only as a substrate for protein synthesis. In analogy, decreased glutamine concentrations were detected after intakes of more than 2 g protein/kg/d as well as in catabolic patients [19, 32, 33]. Interestingly, strong negative correlations were reported between body fat content and glutamine/cystine ratios of healthy persons [19] which corresponds with data of our subjects (*r* = −0.517, *P* < .0003). Although, the biological or metabolic significance of this effect cannot be explained sufficiently it has been reported that catabolic conditions may result in lower plasma concentrations of glutamine or glutamine/cystine ratios [19, 32]. 

Finally, we detected similar THC as well as methionine concentrations 4 weeks after exposure to either M or NOM. Methionine is known as the sole precursor of THC. However, the calculated methionine intake has been considerably lower during NOM as compared to M (Table 2). The results agree with those showing that a high-methionine high-protein diet through increasing lean meat consumption in overweight subjects does not raise THC concentrations as compared with low-methionine low-protein diets after 6 months [34]. Therefore, the results may indicate no increasing risk of M compared to NOM for cardiovascular diseases based on THC as it have been shown for other unbalanced diets [35].

Limitations to our research include the fact that meat is not only a source of dietary protein. Among other nutrients it was proposed that iron derived from red meat may increase iron stores and consequently, could initiate oxidative damage and inflammation. This was suggested to be responsible for long-term adverse effects resulting in coronary heart disease and type 2 diabetes. However, a replacement of dietary carbohydrate with protein from red meat by an addition of lean red meat of approximately 215 g/d was not shown to elevate several parameters responsible for oxidative stress and inflammation after an exposure of 8 weeks [36]. In addition, high-meat intake can be associated with higher saturated fat intake. Therefore, it was not surprising that we computed significantly higher-fat and -energy intakes during M compared to NOM. However, we find it meaningful that these modifications did not increase the body fat content and blood lipid parameters during M compared to NOM. Furthermore, it is not possible to generalize our results because we recruited only young healthy women for this study and one cannot exclude effects of sex and age as well. Nonetheless, our results and conclusions of this study are limited to the short-term because subjects were exposed only for 4 weeks. Long-term studies are required to define specific mechanisms and to explain the benefits or risks of high-meat intakes. 

In summary, a 4 weeks exposure to 200 g lean meat added to the habitual diet of healthy young woman resulting in high-protein intakes seems not to produce adverse effects on body composition, concentrations of blood lipids, insulin, glucose, and THC compared with an exclusion of all meat products. The additional meat consumption may be effective in enhancing FFM with no change in fat mass. The urinary 3MH excretion may serve as a reliably biomarker for the consumption of meat protein provided that the validity was assessed in further studies. Further research is needed to assess the specific mechanisms that explain the possible benefits or risks of long-term high-protein (meat) consumption for nutrition-dependent diseases.

## Figures and Tables

**Table 1:** Characteristics of women at baseline and after 4 weeks additional meat intake (M, 200 g pork fillet/d) or exclusion of meat products (NOM).^1^

		Baseline	M	NOM
Age	(y)	26.1 ± 2.0	—	—
Weight	(kg)	60.4 ± 5.5	60.7 ± 5.8^b^	60.1 ± 5.7^a^
Height	(cm)	167.4 ± 6.3	—	—
BMI	(kg/m^2^)	21.6 ± 2.0	21.7 ± 2.0^b^	21.5 ± 2.0^a^
Body fat mass	(kg)	16.2 ± 4.7	16.2 ± 5.0	16.1 ± 4.9
Fat-free body mass	(kg)	44.2 ± 4.8	44.5 ± 4.5	44.0 ± 4.4

^1^ Values are means ± SD, *n* = 14. The values at baseline did not differ significantly between subjects starting intervention with either M or NOM. ^a,b^Within a row different superscripts indicate *P* < .05 between M and NOM (paired *t*-test).

**Table 2:** Nutrient intakes of women at baseline and during 4 weeks periods of consuming diets with either additional meat content (M, 200 g pork fillet/d) or exclusion of meat products (NOM).^1^

		Baseline	M	NOM
Total protein	(g/kg·d)	1,40 ± 0,29	2,25 ± 0,35^b^	1,15 ± 0,26^a^
	(g/d)	84,1 ± 16,0	134,7 ± 15, 8^b^	69,3 ± 16, 5^a^
	(% of energy intake)	17.1 ± 3.2	25.2 ± 3.0^b^	14.0 ± 3.4^a^
Leucine	(g/d)	6.51 ± 1.33	9.86 ± 1.32^b^	5.44 ± 1.32^a^
Methionine	(g/d)	1.74 ± 0.39	2.99 ± 0.36^b^	1.32 ± 0.34^a^
Vegetable protein	(g/d)	29.7 ± 5.9	30.1 ± 8.5	32.1 ± 6.4
Animal protein	(g/d)	54.4 ± 16.1	104.5 ± 15.3^b^	37.1 ± 12.7^a^
Fat	(g/d)	71,9 ± 13,1	81,3 ± 16, 4^b^	69, 8 ± 14, 2^a^
	(% of energy intake)	32.4 ± 5.9	33.0 ± 6.8	33.2 ± 6.8
Carbohydrates	(g/d)	253,8 ± 50,0	229,5 ± 47,3	248,4 ± 40,5
	(% of energy intake)	51.0 ± 10.2	42.0 ± 8.8	53.3 ± 8.7
Energy	(kcal/d)	2062 ± 330	2247 ± 305^b^	1956 ± 328^a^

^1^ Values are means ± SD, *n* = 14. The dietary intakes at baseline did not differ significantly between subjects starting intervention with either M or NOM. ^a,b^Within a row different superscripts indicate *P* < .05 between M and NOM (paired *t*-test).

**Table 3:** Plasma metabolite concentrations, urinary excretion of nitrogen, urea, and 3-methylhistidine of women at baseline and after 4 weeks periods of either additional meat consumption (M, 200 g pork fillet/d) or exclusion of meat products (NOM).^1^

		Baseline	M	NOM
*Plasma concentration*				
Urea	(mmol/L)	3.96 ± 1.22	5.32 ± 1.03^b^	3.48 ± 0.65^a^
Total cholesterol	(mmol/L)	4.86 ± 0.93	4.59 ± 0.87	4.84 ± 0.81
HDL-cholesterol	(mmol/L)	1.65 ± 0.24	1.61 ± 0.23	1.62 ± 0.21
LDL-cholesterol	(mmol/L)	2.75 ± 0.75	2.60 ± 0.57	2.72 ± 0.6
Triacylglycerol	(mmol/L)	1.00 ± 0.27	0.84 ± 0.23^a^	1.10 ± 0.35^b^
NEFA	(mmol/L)	0.36 ± 0.17	0.31 ± 0.12	0.35 ± 0.18
Glucose	(mmol/L)	4.65 ± 0.37	4.46 ± 0.31	4.56 ± 0.28
Insulin	(mU/L)	6.09 ± 3.54	6.17 ± 2.7	5.07 ± 1.46
Total homocysteine	(*μ*mol/L)	6.93 ± 1.50	9.77 ± 2.75	9.91 ± 1.74
3-Methylhistidine	(*μ*mol/L)	3.20 ± 0.70	5.80 ± 1.7^b^	2.60 ± 0.4^a^
*Urinary excretion*				
Nitrogen	(g/d)	10.1 ± 2.8	15.9 ± 3.7^b^	8.8 ± 2.2^a^
Urea	(mmol/d)	296 ± 77	440 ± 105^b^	248 ± 74^a^
3-Methylhistidine	(*μ*mol/d)	229 ± 93	427 ± 99^b^	133 ± 41^a^

^1^ Values are means ± SD, *n* = 14. The dietary intakes at baseline did not differ significantly between subjects starting intervention with either M or NOM. ^a,b^Within a row different superscripts indicate *P* < .05 between M and NOM (paired *t*-test).

**Table 4:** Changes of plasma metabolite concentrations and urinary excretion of urea and of 3-methylhistidine of women during 4 weeks periods of either additional meat consumption (M, 200 g pork fillet/d) or exclusion of meat products (NOM).^1^

		M	NOM
*Plasma concentration*			
Urea	(mmol/L)	+1.59 ± 1.16***	−1.41 ± 1.23***
Total cholesterol	(mmol/L)	−0.35 ± 0.35**	+0.14 ± 0.64
HDL-cholesterol	(mmol/L)	−0.05 ± 0.15	0.00 ± 0.17
LDL-cholesterol	(mmol/L)	−0.24 ± 0.34*	−0.05 ± 0.53
Triacylglycerol	(mmol/L)	−0.14 ± 0.30	+0.20 ± 0.42
NEFA	(mmol/L)	−0.04 ± 0.13	0.00 ± 0.16
Glucose	(mmol/L)	−0.25 ± 0.34*	+0.12 ± 0.36
Insulin	(mU/L)	−0.23 ± 3.31	−0.63 ± 3.12
Total homocysteine	(*μ*mol/L)	+1.43 ± 1.23***	+2.29 ± 2.03***
3-Methylhistidine	(*μ*mol/L)	+3.00 ± 1.70***	−2.20 ± 2.00***
*Urinary excretion*			
Urea	(mmol/d)	+160 ± 95***	−156 ± 155**
3-Methylhistidine	(*μ*mol/d)	+274 ± 109***	−234 ± 147***

^1^ Values are means ± SD, *n* = 14. Subject specific changes were calculated as difference after M or NOM in relation to values before M or NOM. The significant differences from zero (no change) were indicated by ***P < .001, **P < .01, *P < .05 (2-tailed one sample *t*-test with zero as hypothetical mean value).

**Table 5:** Fasting plasma free amino acid concentrations of women at baseline and after 4 weeks periods of either additional meat consumption (M, 200 g pork fillet/d) or exclusion of meat products (NOM).^1^

	Baseline	M	NOM
*Indispensable amino acids (*μ*mol/L)*			
Histidine	94 ± 10	100 ± 18	98 ± 12
Isoleucine	51 ± 8	53 ± 8	53 ± 7
Leucine	112 ± 18	117 ± 14	119 ± 13
Lysine	159 ± 21	186 ± 34^b^	155 ± 22^a^
Methionine	24 ± 2	28 ± 7	28 ± 3
Phenylalanine	52 ± 8	60 ± 9	56 ± 9
Threonine	153 ± 45	163 ± 50	146 ± 34
Tryptophan	48 ± 8	51 ± 8	51 ± 6
Valine	199 ± 39	233 ± 39^b^	204 ± 23^a^
*Dispensable amino acids (*μ*mol/L)*			
Alanine	331 ± 61	371 ± 95	455 ± 136
Arginine	73 ± 20	80 ± 17	77 ± 14
Asparagine	56 ± 6	61 ± 10	61 ± 7
Cystine	50 ± 6	53 ± 7	53 ± 9
Glutamine	552 ± 72	531 ± 63	567 ± 72
Glutamic acid	18 ± 5	20 ± 7	20 ± 8
Glycine	195 ± 62	194 ± 58	228 ± 77
Proline	159 ± 41	165 ± 46	185 ± 40
Serine	96 ± 15	96 ± 14	104 ± 17
Tyrosine	47 ± 12	57 ± 15	55 ± 9

^1^ Values are means ± SD, *n* = 14. The dietary intakes at baseline did not differ significantly between subjects starting intervention with either M or NOM. ^a,b^Within a row different superscripts indicate *P* < .05 between M and NOM (paired *t*-test).

## Abbreviations

3MH: 3-methylhistidine
BMI: Body mass index
FFM: Fat-free body mass
M: 4 weeks period of habitual diet with additional 200 g pork fillet per d
NEFA: Nonesterified fatty acids
NOM: 4 weeks period of habitual diet with exclusion of meat products
THC: Total homocysteine.
